# Trend in Height of Turkish and Moroccan Children Living in The Netherlands

**DOI:** 10.1371/journal.pone.0124686

**Published:** 2015-05-04

**Authors:** Yvonne Schönbeck, Paula van Dommelen, Remy A. HiraSing, Stef van Buuren

**Affiliations:** 1 TNO Child Health, Leiden, The Netherlands; 2 TNO Life Style, Leiden, The Netherlands; 3 EMGO Institute of Health Care Research, VU University Medical Centre Amsterdam, Amsterdam, The Netherlands; 4 Department of Methodology and Statistics, University of Utrecht, Utrecht, The Netherlands; Centre Hospitalier Universitaire Vaudois, FRANCE

## Abstract

**Objectives:**

To study trends in height of Turkish and Moroccan immigrant children living in The Netherlands, to investigate the association between height and background characteristics in these children, and to calculate height-for-age-references data for these groups.

**Design:**

Nationwide cross-sectional data collection from children aged 0 to 18 years by trained professionals in 1997 and 2009. The study population consisted of 2,822 Turkish 2,779 Moroccan, and 13,705 Dutch origin children in 1997and 2,548 Turkish, 2,594 Moroccan, and 11,255 Dutch origin children in 2009. Main outcome measures: Mean height in cm, and mean height standard deviation scores.

**Results:**

In 2009, mean height at the age of 18y was similar for Turkish and Moroccan children: 177 cm for boys and 163 cm for girls, which was 2 to 3 cm taller than in 1997. Still, Turkish and Moroccan adolescents were 5.5 cm (boys) to 7 cm (girls) shorter than their Dutch peers. No significant differences were found in mean height standard deviation scores across the educational level of the parents, geographical region, primary language spoken at home, and immigrant generation.

**Conclusions:**

While the secular height increase in Dutch children came to a halt, the trend in Turkish and Moroccan children living in The Netherlands continued. However, large differences in height between Turkish and Moroccan children and Dutch children remain. We found no association with the background characteristics. We recommend the use of the new growth charts for children of Turkish and Moroccan origin who have a height-for-age below -2SD on the growth chart for Dutch children.

## Introduction

Secular trends of height have been extensively documented in many populations. However, data on the development of height of immigrant populations over time are scarce. Large differences in height exist between countries and ethnic background [1–4]. Children of immigrants typically differ in height from autochthonous children, as well as from their peers living in their parent’s country of origin [2–5].

The nationwide study in 1997 revealed substantial differences in height between children of Dutch origin and those of Turkish and Moroccan origin living in The Netherlands [3,4]. At the age of 18 years, children of Turkish and Moroccan origin were 9 to 10 cm shorter than their Dutch peers. For this reason, origin-specific growth charts for these groups have been made available. These charts can be used to evaluate height of children of Turkish or Moroccan origin who are considered short on the regular Dutch growth references, which are based on children of Dutch origin. Since 1997, the height of children of Dutch origin has not increased any further [6]. It is not yet known if this is also the case in children of Turkish and Moroccan origin. If height of these children converges towards the Dutch growth patterns, the question arises whether origin-specific charts are still needed.

Given the importance of environmental factors on human growth [7,8], the height of immigrants is expected to converge to the height of the hosting population. Very little is known about the speed of this process, and which factors influence it. The speed of the process is likely to depend in the amount of social acculturation. We would, therefore, expect to see smaller height differences with the autochthonous population in immigrant families with a faster adaptation to the country in which they live.

In this paper, we describe the trend in height since 1997 of children of Turkish and Moroccan origin living in The Netherlands. We compare their height with the height of Dutch children, and with height of children living in Turkey and Morocco. Furthermore, we investigate the association between height and the educational level of the parents, geographical region, primary language spoken at home, and immigrant generation. We present new height-for-age reference data for 0 to 18 year olds of Turkish and Moroccan origin.

## Methods

### Ethics statement

Data collection of growth studies is part of routine youth health care in The Netherlands, and is not regarded as medical research [9]. In the Dutch nationwide surveys, written informed consent was not needed. Verbal consent was obtained from each child (and/or parent for children younger than 16 years). Cooperation, or lack thereof, was registered on the questionnaire. The data were analyzed anonymously. The Medical Ethical Review Board of Leiden University Medical Center approved of the study and the way consent was obtained.

### Study population

Cross-sectional growth data of Dutch, Turkish and Moroccan children aged 0 to18 years and living in The Netherlands were collected within the Fourth Dutch Growth Study from 1997 and the Fifth Dutch Growth Study from 2009. Origin was defined according to the country of birth of the parents [10]: Dutch if both parents were born in The Netherlands; Turkish if the mother was born in Turkey, or if the mother was born in The Netherlands and the father in Turkey; Moroccan if the mother was born in Morocco, or if the mother was born in The Netherlands and the father in Morocco. Data were obtained at Well Baby Clinics, Municipal Health Services (MHS), schools and a festival. To obtain sufficient data of Turkish and Moroccan children, oversampling was done in the four major cities Amsterdam, Rotterdam, The Hague and Utrecht, where most Turkish and Moroccan children in The Netherlands live. In 2009, the MHS in Amsterdam and The Hague supplemented the sample with existing growth data of Turkish and Moroccan children (n = 910, and n = 1,529). The methodology of the growth studies was similar, with the objective to allow comparison over time. For more detail, see the original publications [3,4,11–13].

### Exclusion criteria

The exclusion criteria for both studies were similar. Children with diagnosed growth disorders and those on medication known to interfere with growth were excluded. Children with an origin other than Dutch, Turkish or Moroccan were excluded.

### Measurements

The measurements were standardised and were performed by trained health care professionals. Infants’ length was measured to the nearest 0.1 cm in the supine position until two years of age. From around two years of age, standing height was measured to the nearest 0.1 cm. Demographic characteristics of the children were obtained from the children or their parents by health care professionals by means of a questionnaire.

### Variable definitions

The sample was divided into two geographical regions: major cities (Amsterdam, Rotterdam, Utrecht, and The Hague) and non-major cities (all other regions). Educational level of the parents was defined as the educational level achieved by the highest educated parent, and categorized into low, middle, and high [14] (not available for the supplemented sample of Turkish and Moroccan children from The Hague in 2009). Primary language spoken at home was classified as Dutch or non-Dutch, and immigrant generation as 1st/2nd generation or 3rd generation. Third generation Turkish and Moroccan children were Dutch children (both parents born in The Netherlands) with at least one grandparent born in Turkey or Morocco, respectively. Data on language and generation were only available for 2009 and not for the supplemented samples of Turkish and Moroccan children from The Hague and Amsterdam.

### Statistical analyses

Data were cleaned using descriptive statistics including frequency tables, contingency tables and scatter plots. Standard deviation scores (SDS) [15] per age were calculated using the 1997 origin-specific height references [3,4,11]. Outliers, defined as values over +5 SDS or below -5 SDS were checked for data entry errors and corrected. If no correction was possible, these measurements were considered erroneous and defined as missing. The difference between length and height in Dutch children in 1997 was 0.4cm [11]. In daily practice, the transition from length to height measurement depends on the age at which a child can properly stand up straight. This makes a ‘smooth joint’ at the age of two years the preferred way to handle the difference between length and height. No adjustments of the data were necessary to obtain a good fit of the data.

Three separate sets of reference values were used to define SDS. SDS based on the 1997 origin-specific height references were only used for data cleaning. To compare height SDS across origin and cross generations in 2009, SDS were calculated using the 2009 Dutch height references [6]. For all other comparisons, the origin-specific height references presented in this paper were used for SDS calculation.

Reference values for height-for-age in 2009 were calculated using the LMS method [16]. The LMS method summarizes the SDS lines by three smooth curves representing skewness (L curve), the median (M curve), and coefficient of variation (S curve). L values of 1 indicate normality and smaller values represent progressively greater skewness. The M curve is the 0 SDS line or 50th centile curve. The S curve defines the coefficient of variation. The choice of the smoothing parameters (effective degrees of freedom, edf’s) for the L, M, and S curves was made by creating worm plots: local detrended QQ plots of the SDS of the reference sample across 16 age groups [17]. The curves were fitted as cubic splines. Finally, the age-related reference values were estimated. In case of a normal distribution, so if L = 1, the reference values can be summarized by the mean and standard deviation (SD) per age.

Unadjusted (differences in) mean height and mean height SDS are presented. Linear regression was used to test the association of height SDS in 2009 with geographical region, educational level of the parents, primary language spoken at home and generation for Turkish and Moroccan children, corrected for sex, age, and age squared. P-values < 0.05 (two-sided) were considered statistically significant.

R version 2.9.0 with GAMLSS-package was used for the imputation and for estimating the height SDS reference values [18]. All other statistical analyses were performed in IBM SPSS Statistics version 20.0 for Windows.

## Results

Table 1 presents baseline characteristics for the 1997 and 2009 populations. The Dutch samples were representative for the Dutch population in terms of geographical region and educational level of the child [11,12]. The Turkish and Moroccan samples were oversampled in the major cities, with 100% living in the major cities in the 1997 sample, and 87% in the 2009 sample.

**Table 1:** Background characteristics of the study populations in 1997 and 2009.

		Turkish				Moroccan				Dutch			
		1997		2009		1997		2009		1997		2009	
		(n = 2,822)		(n = 2,548)		(n = 2,779)		(n = 2,594)		(n = 13,705)		(n = 11,255)	
		n	%	n	%	n	%	n	%	n	%	n	%
Sex	boys	1,459	51.7	1,296	50.9	1,412	50.8	1,270	49.0	7,044	51.4	5,434	48.3
	girls	1,363	48.3	1,252	49.1	1,367	49.2	1,324	51.0	6,661	48.6	5,821	51.7
Age	0–4 years	938	33.2	935	36.7	915	32.9	915	35.3	5,737	41.9	4,703	41.8
	5–11 years	890	31.5	1,112	43.6	875	31.5	1,222	47.1	3,001	21.9	3,409	30.3
	12–18 years	994	35.2	501	19.7	989	35.6	457	17.6	4,967	36.2	3,143	27.9
Parental education	low	2,045	72.5	551	21.6	2,110	75.9	513	20.1	4,042	29.5	1,638	14.5
	Medium	244	8.6	351	13.8	71	2.6	318	12.3	4,562	33.3	3,829	34.0
	high	53	1.9	174	6.8	33	1.2	175	6.7	4,485	32.7	4,841	43.0
	unknown	480	17.0	1,472	57.8	565	20.3	1,578	60.8	616	4.5	947	8.4
Region	major city	2,815	99.8	2,154	84.5	2,764	99.5	2,301	88.7	1,398	10.2	2,112	18.8
	outside city	5	0.2	394	15.5	13	0.5	293	11.3	12,265	89.5	9,146	81.2
	unknown	2	0.1	-	-	2	0.1	-	-	42	0.3	-	-
Language	Dutch	-	-	313	12.3	-	-	612	23.6	-	-	8,907	79.1
	other	-	-	947	37.2	-	-	679	26.2	-	-	71	0.6
	unknown	-	-	1,288	50.5	-	-	1,303	50.2	-	-	2,277	20.2
Third generation**		-	-	50	-	-	-	-	44	-	-	-	-

* Including 1,801 imputed cases

** These children are included in the Dutch sample and only categorized as Turkish or Moroccan for the analyses regarding immigrant generation. Therefore no proportion was calculated.

### Height of Turkish and Moroccan children in The Netherlands in 2009

Table 2 shows the new reference values for height-for-age (mean height and SD per age) of Turkish and Moroccan boys and girls living in The Netherlands in 2009. Height-for-age was normally distributed. Final height in girls was reached at the age of 16 (Moroccan) and 17 (Turkish) years. Height at the age of 18y was similar for Turkish and Moroccan adolescents: around 177 cm for boys and almost 163 cm for girls. SD estimates were higher in adolescents of Moroccan origin.

**Table 2:** Reference values for height-for age: mean height and standard deviation (SD) of Turkish and Moroccan boys and girls in The Netherlands in 2009.

	Turkish				Moroccan			
	Boys		Girls		Boys		Girls	
	(n = 1,296)		(n = 1,252)		(n = 1,270)		(n = 1,324)	
age	mean	SD	mean	SD	mean	SD	mean	SD
(years)	(cm)	(cm)	(cm)	(cm)	(cm)	(cm)	(cm)	(cm)
*0.0767	54.9	2.0	53.5	2.3	54.4	2.0	52.9	2.0
0.25	60.8	2.2	59.2	2.3	60.2	2.2	58.9	2.1
0.50	68.0	2.4	66.1	2.4	67.1	2.4	65.8	2.3
0.75	73.2	2.5	71.3	2.5	72.1	2.6	71.1	2.5
1.00	77.3	2.7	75.6	2.5	76.1	2.8	75.0	2.6
1.25	80.7	2.8	79.3	2.6	79.5	3.0	78.1	2.8
1.50	83.6	3.0	82.6	2.7	82.4	3.1	80.9	2.9
1.75	86.2	3.1	85.3	2.9	85.1	3.2	83.7	3.0
2.00	88.6	3.1	87.8	3.0	87.7	3.3	86.5	3.1
3.00	97.4	3.5	96.3	3.4	96.8	3.7	96.0	3.6
4.00	105.6	3.9	104.0	3.9	104.5	4.0	103.5	3.9
5.00	112.6	4.4	110.9	4.3	111.4	4.4	110.2	4.2
6.00	118.3	4.8	117.0	4.6	117.7	4.8	116.8	4.5
7.00	123.6	5.2	122.8	5.0	123.9	5.2	123.0	4.9
8.00	129.5	5.8	128.1	5.4	129.4	5.7	128.5	5.4
9.00	135.3	6.3	133.9	5.8	134.5	6.1	134.1	6.1
10.00	140.6	6.8	140.5	6.2	140.1	6.6	140.2	6.9
11.00	145.9	7.2	147.5	6.4	145.4	7.2	147.0	7.3
12.00	151.9	7.6	153.2	6.4	150.5	7.7	152.9	7.3
13.00	158.2	7.8	156.9	6.3	156.3	8.1	157.4	7.0
14.00	164.0	7.7	159.4	6.2	163.3	8.3	160.5	6.7
15.00	168.7	7.5	160.9	6.1	169.9	8.1	162.4	6.5
16.00	172.7	7.2	161.9	6.0	174.0	7.9	162.8	6.5
17.00	175.4	6.9	162.6	6.0	176.1	7.8	162.8	6.5
18.00	176.8	6.8	162.6	6.0	177.2	7.7	162.8	6.5

*0.0767 = 4 weeks

No significant differences were found in mean height SDS of Turkish or Moroccan children living in the major cities versus those living outside of these cities. In Turkish children, mean height SDS was slightly higher among those of higher educated parents, but this was not statistically significant. No such trend was seen in Moroccan children. Mean height SDS of Turkish and Moroccan children who speak Dutch at home did not differ from that of children from non-Dutch speaking families. Of the small group of third generation children (n = 50 for Turkish, n = 44 for Moroccan origin), 74% was under the age of two years, and 87% was under four years of age. We, therefore, compared height across generations in children under four years of age. Mean height of Turkish and Moroccan third generation children was respectively 0.02 SDS lower and 0.14 SDS higher compared to children from the first and second generation, but this was not statistically significant.

### Height of Turkish and Moroccan children in 1997 and 2009

**Fig 1:**
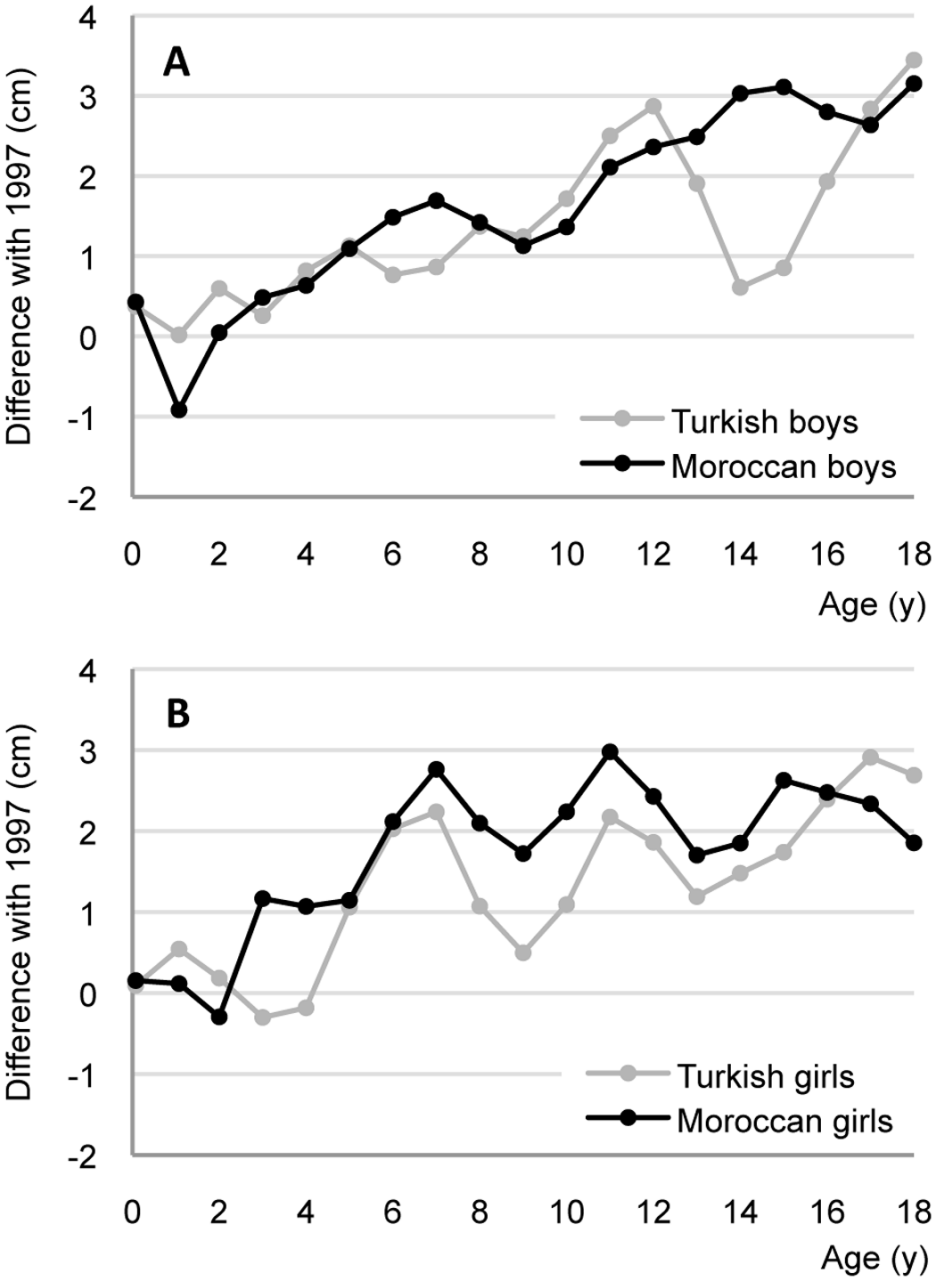
Height difference with 1997 (horizontal line at 0 cm) of Turkish and Moroccan boys (A) and girls (B) aged 0–18 y in 2009.

Fig 1 compares the height difference between 2009 and 1997 in cm per age of Turkish and Moroccan boys (A) and girls (B). From the age of one year (Turkish) and two years (Moroccan) onward, the boys were taller than in 1997, reaching a difference of 3.2 to 3.5 cm at the age of 18 years. In girls, we saw an increase in height compared to 1997 from the age of two years (Moroccan) and four years (Turkish) onwards. At the age of 18 years, girls were 1.9 (Moroccan) to 2.7 (Turkish) cm taller than in 1997. These data correspond to a positive trend in final height of 2.8 cm/decade for Turkish and Moroccan boys and 1.9 cm/decade for Turkish and Moroccan girls.

### Height of Turkish and Moroccan children versus Dutch children in 2009

**Fig 2:**
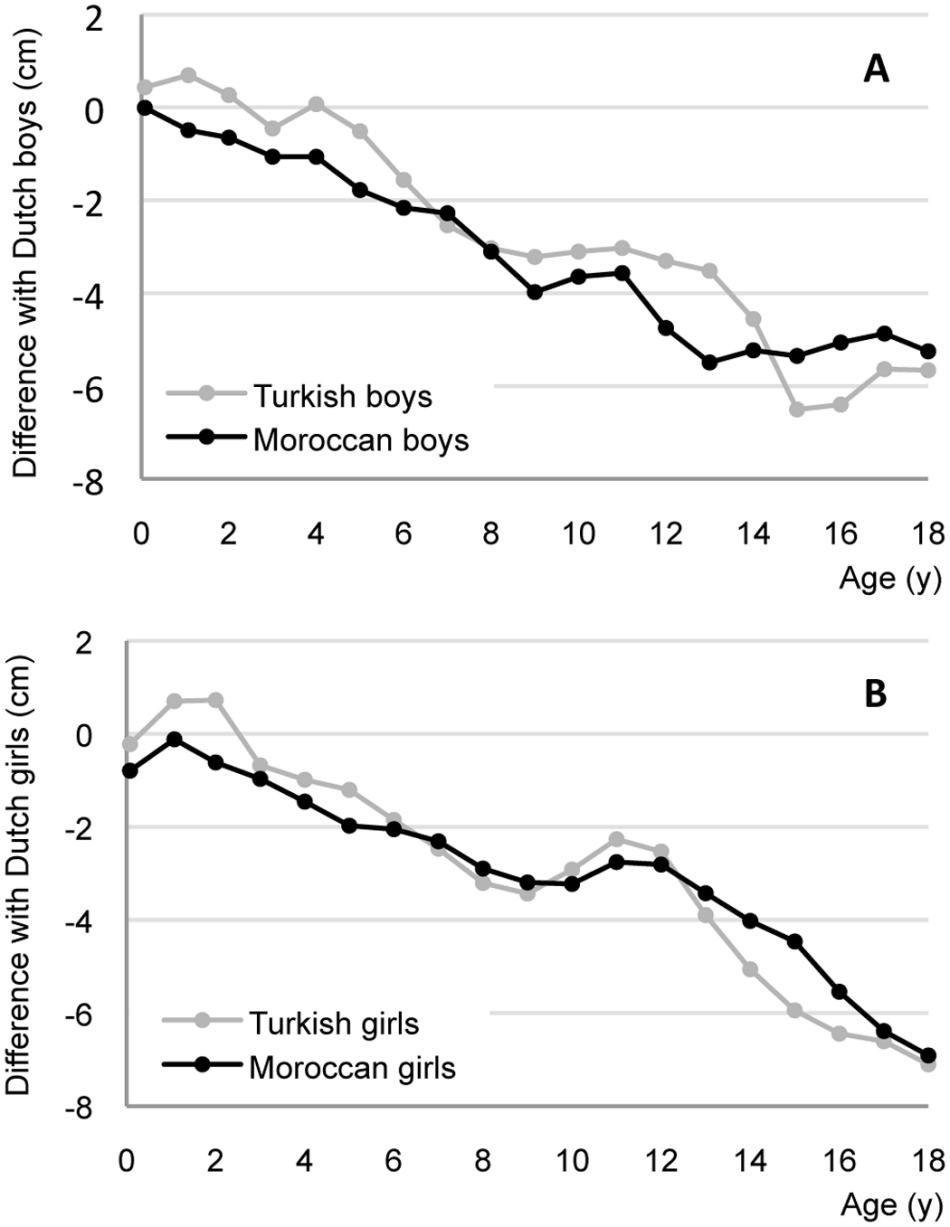
Height difference with Dutch children (horizontal line at 0 cm) of Turkish and Moroccan boys (A) and girls (B) aged 0–18 y in 2009.

Fig 2 shows the height difference in cm between Dutch children (horizontal line at 0 cm) and Turkish and Moroccan children in 2009. Overall, we saw lower growth rates in Turkish and Moroccan children compared to Dutch children from the age of one year onwards. Moroccan boys were shorter from birth onwards, and Turkish boys from the age of four years onwards. Similarly, Moroccan girls were shorter than their Dutch peers from birth, while Turkish girls began to diverge from the Dutch from two years of age. At the age of 18 years, the height differences reached around -5.5 cm in boys and -7.0 cm in girls. The ‘bumps’ between the age of 9 and 11 years in the downward line reflect the faster progression through puberty in Turkish and Moroccan children compared to the Dutch [4,19,20].

Third generation Turkish and Moroccan children were respectively 0.02 and 0.13 SDS shorter than the Dutch children under four years of age, which was not significantly different, and based on small numbers.

At all levels of parental education, mean height SDS of Dutch children was higher than of Turkish and Moroccan children. The difference between Turkish and Dutch children was largest among those with low educated parents (low: -0.25 SDS, middle: -0.18 SDS, high: -0.12 SDS, p<0.001 for all comparisons). This trend was not seen in Moroccan children (low: -0.34 SDS, middle: -0.40 SDS, high: -0.34 SDS, p<0.001 for all comparisons).

### Comparison with data from WHO and Turkey and Morocco

**Fig 3:**
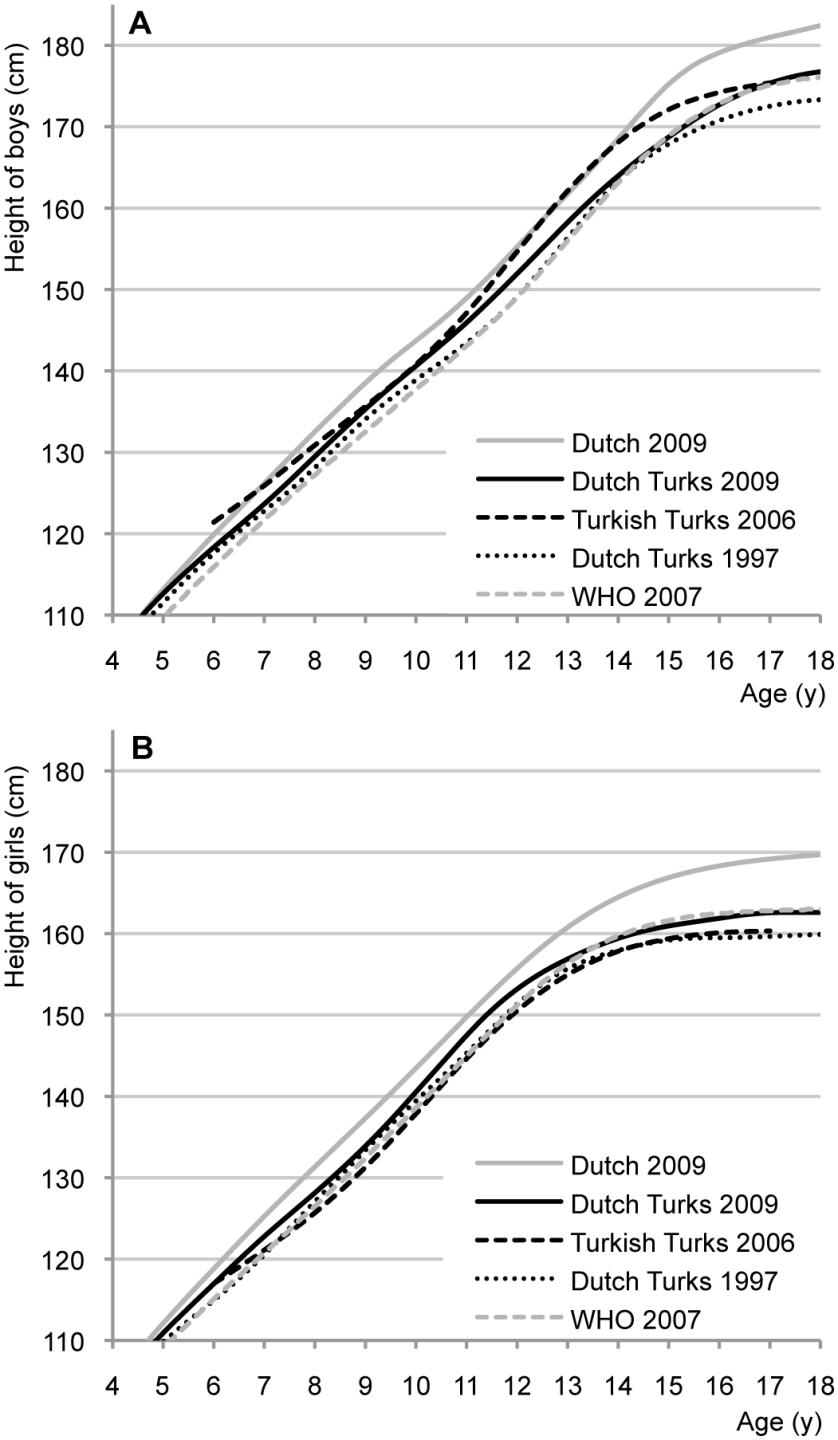
Different height-for-age references for boys (A) and girls (B): Dutch 2009 [6], Dutch Turks 2009, Turkish Turks in Ankara 2006 [22], Dutch Turks 1997 [3], and WHO 2007 [21].

Fig 3 compares mean height-for-age of Dutch, Dutch Turks, Turkish Turks and WHO children [3,6,21,22]. Clear differences exist between Dutch and Turkish children living in The Netherlands in 2009. We also see the trend in height of Turkish children in The Netherlands between 1997 and 2009. The data from Turkey represent height-for-age from 2004–2006 of Turkish children from mixed socioeconomic background living in Ankara [22]. Compared to the Dutch Turks in 2009, the Turkish boys in Turkey were taller at most ages, while the Turkish girls in Turkey were shorter that their Turkish peers in The Netherlands. The final height of Turkish boys in Turkey was similar to that of Turkish boys in The Netherlands in 2009, while the girls in Turkey grew more like the Turkish girls in The Netherlands in 1997. Height of children aged 0–4 years did not show large differences with available data from Turkey, representing children with a high socioeconomic background (data not shown) [3,23]. The final height of the WHO children was similar to the 2009 height of Dutch Turks (and Dutch Moroccans), but before the age of 13/14 years WHO children were considerably shorter.

For Morocco, no recent national growth references were available. One paper from 1995 presented national references, but they were presented as hard to read charts and the exact data were not available on request [24]. From the chart we read a final height of around 174 cm for boys and 161 cm for girls, which is in between the final height in 1997 and 2009 of Moroccan children in The Netherlands.

## Discussion

This study shows a positive trend in height since 1997 in both Turkish and Moroccan children living in The Netherlands. Final height increased in both groups at a rate of 2.8 cm per decade in boys and 1.9 cm per decade for girls. This is remarkable since the secular height increase in Dutch children vanished between 1997 and 2009. The net result is a smaller height difference between Dutch children and Turkish and Moroccan children living in The Netherlands. Still, 18-year old Turkish and Moroccan children are 5.5 cm (boys) to 7 cm (girls) shorter than Dutch children. If the current trends remain, it will take twenty to forty years before the Turkish and Moroccan children catch-up with the height of the Dutch.

Previous studies found that the differences in height between immigrants and indigenous populations could be explained by socioeconomic status rather than by place of birth [25]. In addition, the level of acculturation was found to be associated with the prevalence of overweight, and could possibly also affect height [26–28]. In our study, geographical region, educational level of the parents, primary language spoken at home, and immigrant generation did not explain the difference in height. The height of third generation immigrant children of Moroccan origin lay in between that of the Dutch and second generation Moroccan immigrant children. This could indicate that socioeconomic status and/or acculturation have a positive effect on height, as we know from Dutch registries that immigrants from the second generation are higher educated than those from the first generation [29]. It could also indicate that there are biological effects on growth that may take several generations to overcome, such as low height-for-age of the mother. However, such ‘trend’ across generations was not found in children of Turkish origin, and the differences were not statistically significant. The number of third generation children was too small to draw conclusions. We have to wait for future growth studies to reveal growth differences across generations in more detail, and to determine if growth of the third generation immigrant children converges more towards the height of the Dutch children.

A limitation of our study is that we only had few parameters available, and that the number of missing values was relatively high. Although this was mainly due to the use of supplemented data, rather than parents not wanting to provide the data, we cannot rule out that the missing data biased our results. It would be interesting to include additional parameters to measure acculturation in future (growth) studies, like employment, income, length of stay in The Netherlands, dietary habits, interaction with Dutch society, and values and attitudes towards cultural origin and beliefs.

Turkish and Moroccan immigrants arrived in The Netherlands during the 1970’s and 1980’s for work. The group consisted mainly of lowly educated farmers from rural areas in Turkey and Morocco. Children living in rural areas in Turkey are shorter than children living in Turkey’s larger cities, where the socioeconomic status is generally higher [22,30]. The Turkish children in The Netherlands are substantially taller than the children living in rural areas in Turkey nowadays [30], so the height gain is large. This phenomenon was also seen in other countries[2,5] and is likely to be due to better nutrition, hygiene and health status. Height of Turkish children in The Netherlands from the age of 13 years onwards is now similar to that of children of higher socio-economic background in Istanbul [31]. No proper comparison of height of Moroccan children living in The Netherlands versus Morocco could be made, as recent data from Morocco are lacking.

The observed differences in height between Turkish, Moroccan and Dutch children have implications for clinical practice. The present study documents a considerable increase in height compared to the Turkish and Moroccan population in 1997, which makes the origin-specific charts of 1997 outdated. At the same time, there still was a 5.5 cm to 7 cm difference in final height between the Dutch and Turkish or Moroccan children in 2009. Therefore, using only the Dutch growth charts for Turkish and Moroccan children would classify many children as having short stature, while in fact their height is normal when compared to their ethnic peers. As an alternative, we considered using the WHO references for non-Dutch children who do not grow according to the Dutch references. However, the WHO references lay below the origin-specific references until the age of 13/14 years. Consequently, using the WHO references could result in missing-Turkish and Moroccan children that would be classified as short stature on the origin-specific references. We therefore decided to update the origin specific growth charts.

We recommend using the Dutch growth charts for all children in The Netherlands, and the origin-specific growth charts for Turkish and Moroccan children who have a height-for-age below -2SD on the Dutch growth charts. For children of other origins living in The Netherlands who are short relative to the Dutch references, we recommend using the WHO references as a fall back.

Future growth studies aiming at height of Turkish and Moroccan children are important to monitor the development of the trend in height of these children. The growth charts for Dutch, Turkish, and Moroccan children are available at www.tno.nl/growth.

## Supporting Information

S1 Dataset Height Data from the Dutch Growth Studies in 1997 and 2009. (SAV)
